# Modelling the effective reproduction number of vector-borne diseases: the yellow fever outbreak in Luanda, Angola 2015–2016 as an example

**DOI:** 10.7717/peerj.8601

**Published:** 2020-02-27

**Authors:** Shi Zhao, Salihu S. Musa, Jay T. Hebert, Peihua Cao, Jinjun Ran, Jiayi Meng, Daihai He, Jing Qin

**Affiliations:** 1 School of Nursing, Hong Kong Polytechnic University, Hong Kong, China; 2 Division of Biostatistics, JC School of Public Health and Primary Care, Chinese University of Hong Kong, Hong Kong, China; 3 Clinical Trials and Biostatistics Lab, Shenzhen Research Institute, Chinese University of Hong Kong, Shenzhen, China; 4 Department of Applied Mathematics, Hong Kong Polytechnic University, Hong Kong, China; 5 Department of Hepatobiliary Surgery II, Zhujiang Hospital, Southern Medical University, Guangzhou, China; 6 School of Public Health, Li Ka Shing Faculty of Medicine, University of Hong Kong, Hong Kong, China; 7 School of Economics and Finance, Xi’an International Studies University, Xi’an, China

**Keywords:** Reproduction number, Vector-borne disease, Epidemic, Mathematical modelling, Yellow fever, Angola, Luanda

## Abstract

The burden of vector-borne diseases (Dengue, Zika virus, yellow fever, etc.) gradually increased in the past decade across the globe. Mathematical modelling on infectious diseases helps to study the transmission dynamics of the pathogens. Theoretically, the diseases can be controlled and eventually eradicated by maintaining the effective reproduction number, (${\mathcal{R}}_{\mathrm{eff}}$), strictly less than 1. We established a vector-host compartmental model, and derived (${\mathcal{R}}_{\mathrm{eff}}$) for vector-borne diseases. The analytic form of the (${\mathcal{R}}_{\mathrm{eff}}$) was found to be the product of the basic reproduction number and the geometric average of the susceptibilities of the host and vector populations. The (${\mathcal{R}}_{\mathrm{eff}}$) formula was demonstrated to be consistent with the estimates of the 2015–2016 yellow fever outbreak in Luanda, and distinguished the second minor epidemic wave. For those using the compartmental model to study the vector-borne infectious disease epidemics, we further remark that it is important to be aware of whether one or two generations is considered for the transition “from host to vector to host” in reproduction number calculation.

## Introduction

Vector-borne disease epidemics pose a serious threat to global health. Especially in tropical and sub-tropical regions, most vector-borne diseases are treated as a part of neglected tropical diseases (NTDs) (Fenwick, 2012), present endemic features, and are persistent in the interface of host and vector communities. During 2014, the historical large-scale dengue fever caused an extensive international epidemic in southern China as well as other regions in Southeastern Asia (Sang et al., 2015; Stanaway et al., 2016). The Zika virus (ZIKV) emerged in the Pacific area in 2008 (Duffy et al., 2009; Besnard et al., 2014; Cauchemez et al., 2016), then in South America since 2015, and caused more than 500,000 (confirmed or probable) cases, while the true number of cases remains unclear (He et al., 2017; Gao et al., 2016; Johansson et al., 2016; Ferguson et al., 2016; He et al., 2019). The West Nile virus (WNV) was widespread across tropical parts globally, and was introduced into North America in 1999, which lead to an approximated 1.8 million infections from 1999 to 2010 (Kilpatrick, 2011). The chikungunya virus (CHIKV) hit the Americas and beyond, where tens of millions of previously unexposed persons would be at risk (Fischer & Staples, 2014; Weaver & Lecuit, 2015). In 2015–2016, the largest yellow fever (YF) outbreak (since the 1980s) occurred in Angola and the Democratic Republic of the Congo (DRC) (Zhao et al., 2018b; Kraemer et al., 2017; Wu et al., 2016; Shearer et al., 2017). After Africa, the YF continuously posed a serious threat to the unprotected population of southern Brazil, which was believed to have eradicated YF after the middle of the last century (Barrett, 2018). Malaria epidemics occur from time to time in many under-developed places in the tropical and sub-tropical regions (Caminade et al., 2014; Murray et al., 2012). The increasing frequency of such outbreaks over the past decades urges disease control and prevention studies (Johansson et al., 2012; Kraemer et al., 2019).

Mathematical modelling on infectious diseases are developed, and help to study the transmission dynamics of the pathogens from a theoretical point of view (Earn et al., 2008; Brauer & Castillo-Chavez, 2001; Keeling & Rohani, 2011; Grenfell, Dobson & Moffatt, 1995). Theoretically, the infectious diseases can be controlled and finally eradicated by maintaining the effective reproduction number, ${\mathcal{R}}_{\text{eff}}$, strictly less than 1, i.e.,  ${\mathcal{R}}_{\text{eff}}&lt; 1$. The ${\mathcal{R}}_{\text{eff}}$ is the expected number of secondary cases produced by one typical infection joining in a population during its infectious period (Van den Driessche & Watmough, 2002). This theoretical criterion is widely adopted as a threshold to characterise the transmission dynamics and measure the disease control effectiveness (Earn et al., 2008; Keeling & Rohani, 2011). For airborne communicable diseases, e.g., respiratory diseases and most childhood infections, that transmit in the vector-free context, ${\mathcal{R}}_{\text{eff}}$ is given as in Eq. (1).

(1) \begin{eqnarray*}{\mathcal{R}}_{\text{eff}}={\mathcal{R}}_{0}{\mathcal{S}}_{\text{h}},\end{eqnarray*}

where the ${\mathcal{R}}_{0}$ is the basic reproduction number, and the ${\mathcal{S}}_{\text{h}}$ is the susceptibility in the human population. The ${\mathcal{R}}_{0}$ is defined as the expected number of secondary cases produced by one typical infection joining in a completely susceptible population during its infectious period (Heffernan, Smith & Wahl, 2005). This formula in Eq. (1) has been well-studied and wildly used to quantify the transmissibility of infectious diseases (Earn et al., 2008; Brauer & Castillo-Chavez, 2001; Keeling & Rohani, 2011; Grenfell, Dobson & Moffatt, 1995).

We used the recent yellow fever outbreak in Luanda, Angola from 2015 to 2016 as an example to implement the ${\mathcal{R}}_{\text{eff}}$, and demonstrate the difference(s) between different forms of the ${\mathcal{R}}_{\text{eff}}$s of airborne diseases and vector-borne diseases. The YF outbreak included 941 reported cases with 73 deaths in Luanda, the capital city of Angola from December 2015 to June 2016 (Zhao et al., 2018b; WHO, 2017). The local authority had conducted large-scale mass vaccination campaign since February 2016, and the vaccine program immunised approximated 55% of the local population within 6 months since started (Zhao et al., 2018b; WHO, 2017). Owing to the timely and large-scale mass vaccination campaign, it was estimated that over 5-fold of both cases and deaths were saved. The YF cases time series in Luanda and the local vaccination coverage were obtained from the situation reports released by the African Health Observatory (AHO) (WHO, 2017).

In this work, we establish a simple (and classic) host-vector compartmental model and derive the effective reproduction number for the transmission dynamics of the vector-borne diseases. We further explore the relationship between the disease control in terms of ${\mathcal{R}}_{\text{eff}}$ and the control efforts of the basic reproduction number (${\mathcal{R}}_{0}$) and population susceptibility. The yellow fever (YF) epidemic in Luanda, Angola from December 2015 to June 2016 is studied as an illustrative example to compare the ${\mathcal{R}}_{\text{eff}}$ estimates in different formulations or approaches.

## Methods

Many vector-borne diseases, such as dengue fever, yellow fever, Zika fever, malaria, etc., are transmitted from vector to host as well as from host to vector. Following previous literature (Earn et al., 2008; Brauer & Castillo-Chavez, 2001; Keeling & Rohani, 2011; Grenfell, Dobson & Moffatt, 1995), the transmission mechanism can be explained by the vector-host epidemic models based on the differential equations, i.e., compartmental models.

### Epidemic model for vector-borne diseases

**Figure 1:**
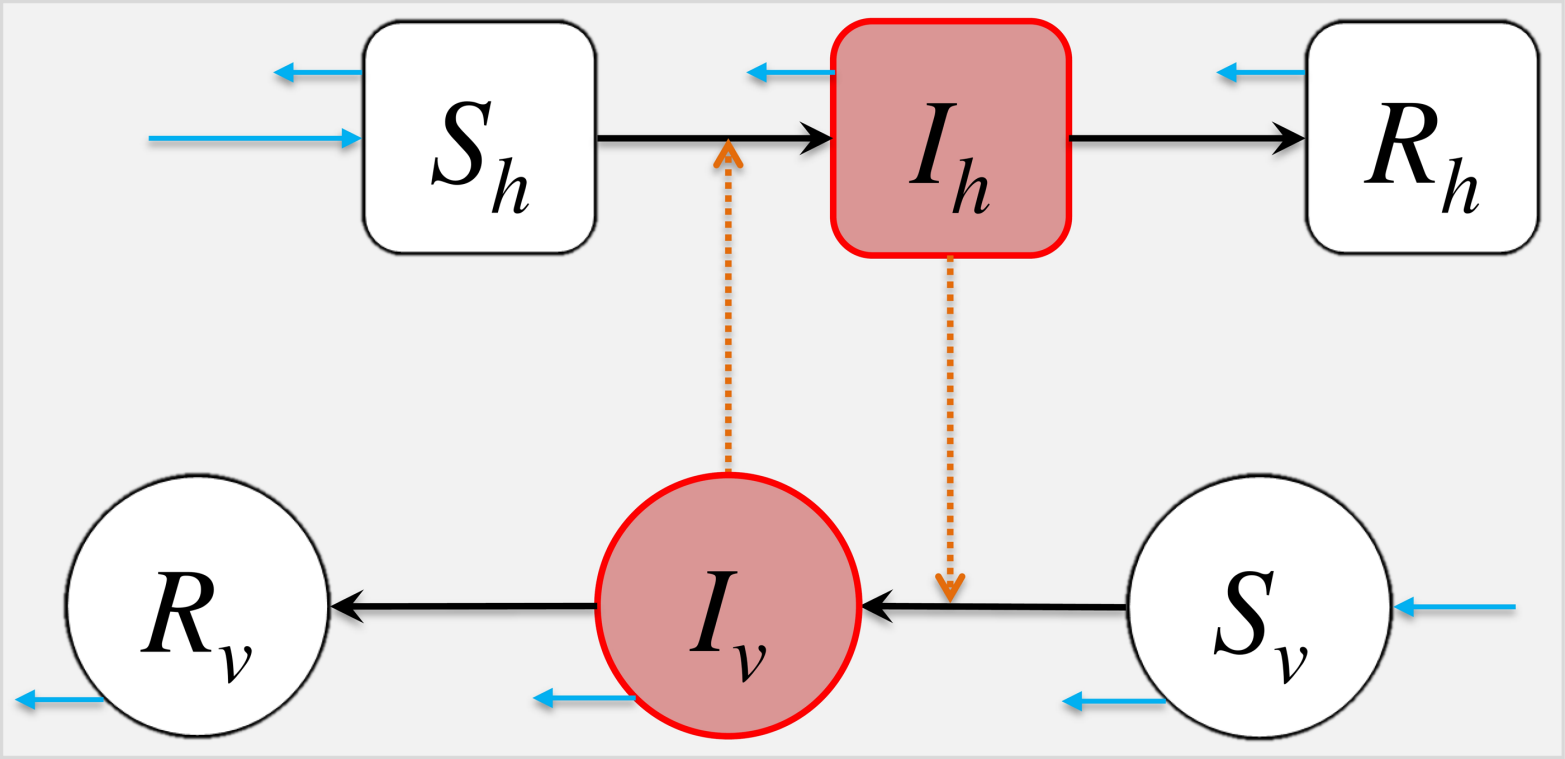
The schematic diagram of model (2). The *S*_*h*_, *I*_*h*_, *R*_*h*_ represent the numbers of susceptible, infected and removed host population. The *S*_*v*_, *I*_*v*_, *R*_*v*_ represent the numbers of susceptible, infected and removed vector population. The black arrows represent the infection status transition paths. The orange dashed arrows represent disease transmission paths, and the light blue arrows represent the natural birth or death of hosts or vectors. Square compartments represent the host classes (or compartments), and circular compartments represent the vector classes. The red compartments represent infected classes.

We adopted the classic “susceptible-infected-removed” (SIR) structural framework to model both host and vector population dynamics (Gao et al., 2016; Tang et al., 2016; Saad-Roy, Van den Driessche & Ma, 2016; Zhao et al., 2018b; Earn et al., 2008; Brauer et al., 2016). We use *S*_*h*_, *I*_*h*_, *R*_*h*_ to denote the numbers of susceptible, infected and removed host population respectively. The *S*_*v*_, *I*_*v*_, *R*_*v*_ denote the numbers of susceptible, infected and removed vector population respectively. The susceptible host becomes infected by the “contact” with infectious vectors, eventually recovers, i.e., move to the recovered class, and remains protected from secondary infection. A similar path is also modelled in the vectors’ population. For simplicity, we ignored the incubation period, commonly denoted by *E*, of the infection in the epidemic model. Based on the above descriptions, the compartmental model is formulated in Eqs. (2). Figure 1 shows the schematic diagram of model (2).

(2) \begin{eqnarray*} \left\{ \begin{array}{@{}l@{}} \displaystyle {S}_{h}^{{^{\prime}}}={\mu }_{h}{N}_{h}-{\beta }_{vh}\cdot \frac{{S}_{h}}{{N}_{h}} {I}_{v}-{\mu }_{h}{S}_{h},\\ \displaystyle {I}_{h}^{{^{\prime}}}={\beta }_{vh}\cdot \frac{{S}_{h}}{{N}_{h}} {I}_{v}-({\gamma }_{h}+{\mu }_{h}){I}_{h},\\ \displaystyle {R}_{h}^{{^{\prime}}}={\gamma }_{h}{I}_{h}-{\mu }_{h}{R}_{h},\\ \displaystyle {S}_{v}^{{^{\prime}}}={B}_{v}(t)-{\beta }_{hv}{S}_{v}\cdot \frac{{I}_{h}}{{N}_{h}} -{\mu }_{v}{S}_{v},\\ \displaystyle {I}_{v}^{{^{\prime}}}={\beta }_{hv}{S}_{v}\cdot \frac{{I}_{h}}{{N}_{h}} -({\gamma }_{v}+{\mu }_{v}){I}_{v},\\ \displaystyle {R}_{v}^{{^{\prime}}}={\gamma }_{v}{I}_{v}-{\mu }_{v}{R}_{v}. \end{array} \right. \end{eqnarray*}

The model parameters are summarised in Table 1, and all parameters are assumed non-negative.

**Table 1:** Summary table of the parameters in model (2).

Parameter	Notation	Unit/Remark
Transmission rate from vector to host	*β*_*vh*_	Host per vector ⋅time
Transmission rate from host to vector	*β*_*hv*_	Per time
Host’s disease-induced removing rate	*γ*_*h*_	Per time
Vector’s disease-induced removing rate	*γ*_*v*_	Per time
Vector’s natural recruiting rate	*B*_*v*_(*t*)	Vector per time
Vector’s natural death rate	*μ*_*v*_	Per time
Host’s natural birth/death rate	*μ*_*h*_	Per time
Vector to host ratio	*m* = *N*_*v*_∕*N*_*h*_	Vector per host
Host’s susceptibility	${\mathcal{S}}_{\text{h}}={S}_{h}{}{N}_{h}$	Unit-free
Vector’s susceptibility	${\mathcal{S}}_{\text{v}}={S}_{v}{}{N}_{v}$	Unit-free

The *N*_*h*_ and *N*_*v*_ are the total numbers of the hosts and vectors respectively. We have

\begin{eqnarray*}{N}_{h}={S}_{h}(t)+{I}_{h}(t)+{R}_{h}(t)=\text{constant}; \text{and} \end{eqnarray*}

\begin{eqnarray*}{N}_{v}(t)={S}_{v}(t)+{I}_{v}(t)+{R}_{v}(t)\not = \text{constant}. \end{eqnarray*}

In our model, *N*_*v*_ = *N*_*v*_(*t*) is time-dependent in a manner that is controlled by the mosquito birth rate *B*_*v*_(*t*), namely ${N}_{v}^{{^{\prime}}}={B}_{v}(t)-{\mu }_{v}{N}_{v}$, which may not be necessarily equal to zero. To guarantee the biological reasonability, the values of all six classes should be non-negative, i.e., ⩾0. Then, *S*_*h*_, *I*_*h*_, *R*_*h*_ ⩽ *N*_*h*_, and *S*_*v*_, *I*_*v*_, *R*_*v*_ ⩽ *N*_*v*_. Although the vector-borne diseases could affect the vector’s lifespan in some occasions, e.g., tick-borne diseases (Niebylski, Peacock & Schwan, 1999), it is conventionally assumed the infected vectors can neither recover nor cause direct mortality in modelling studies, which is true for most of the mosquito-borne diseases, the most common group of vector-borne diseases. For simplicity, the disease-caused vector mortality is neglected in this modelling study. The inclusion of *R*_*v*_ in the model (2) seems unnatural, nevertheless letting *γ*_*v*_ = 0, thus *R*_*v*_ = 0, can resolve this conflict straightforwardly.

Most of the pathogens of the vector-borne diseases merely transmit via two paths, host-to-vector and vector-to-host, e.g., Dengue fever, yellow fever, Chikungunya fever and malaria, etc. For those can be transmitted via host-to-host path, commonly by sexual contact or blood transfusion, e.g., Zika fever, the host-to-host transmission is minor, and dominated by the aforementioned two paths. Therefore, we remark that although the model (2) takes a simple form, it is applicable to model the transmission dynamics of most vector-borne diseases. Model (2) also can be easily extended into more complex form, for instance the model system in Zhao et al. (2018b), Gao et al. (2016) and Tang et al. (2016). As long as the transmission paths remain from-host-to-vector and from-vector-to-host, the relationship between ${\mathcal{R}}_{0}$ and ${\mathcal{R}}_{\text{eff}}$ explored in this paper still holds.

### Basic reproduction number

The basic reproduction number, ${\mathcal{R}}_{0}$, is the expected number of secondary cases produced by one typical infection joining in a **completely susceptible** population during its infectious period (Heffernan, Smith & Wahl, 2005). When ${\mathcal{R}}_{0}&lt; 1$, the disease would die out in the long run. While if ${\mathcal{R}}_{0}&gt; 1$, the disease would spread among the population and may cause a pandemic.

In epidemiology, the next generation matrix is a method used to derive the basic reproduction number, ${\mathcal{R}}_{0}$, for a compartmental model of the spread of infectious diseases. According to Van den Driessche & Watmough (2002), Diekmann, Heesterbeek & Roberts (2009) and Diekmann, Heesterbeek & Metz (1990), a systematic procedure to calculate the ${\mathcal{R}}_{0}$ by solving the dominant eigenvalue, i.e., the eigenvalue with the largest real part, of the next generation matrix, **G**, at the disease-free equilibrium (DFE). Mathematically, it can be shown easily that the DFE exists and is stable for our epidemic model (2). For the next generation matrix **G** = **FV**^−1^, the **F** is the new infection (or transmission) matrix and **V** is the infection transfer (or transition) matrix. The entry of *i*th row and *j*th column of matrix **F** is denoted by *F*_*i*,*j*_, and ${F}_{i,j}= \frac{\partial {\mathcal{F}}_{i}}{\partial {x}_{j}} $ where ${\mathcal{F}}_{i}$ is the *i*th equation of $\mathcal{F}$ and *x*_*j*_ is the *j*th variable of the vector of infected classes. For instance, in Eqs. (2), the *I*_*h*_ and *I*_*v*_ are the infected classes. Similarly, the entry of *i*th row and *j*th column of matrix **V** is denoted by *V*_*i*,*j*_, and ${V}_{i,j}= \frac{\partial {\mathcal{V }}_{i}}{\partial {x}_{j}} $ where ${\mathcal{V }}_{i}$ is the *i*th equation of $\mathcal{V }$ and *x*_*j*_ is the *j*th variable of the vector of infected classes. The vector $\mathcal{F}$ is the transmission rates’ vector quantity, i.e., the changing rates from infected to non-infected classes, and vector $\mathcal{V }$ is the transition rates’ vector quantity, i.e., the changing rates among infected classes. The **F** is the Jacobian matrix of $\mathcal{F}$, and **V** is the Jacobian matrix of $\mathcal{V }$.

For the compartmental model in Eqs. (2), the *I*_*h*_ and *I*_*v*_ are the infected classes, which should be included in the vectors ($\mathcal{F}$ and $\mathcal{V }$) of infected classes. Then, we have $\mathcal{F}= \left( {\scriptsize \begin{array}{@{}c@{}} \displaystyle {\beta }_{vh}\cdot \frac{{S}_{h}}{{N}_{h}} {I}_{v}\\ \displaystyle {\beta }_{hv}{S}_{v}\cdot \frac{{I}_{h}}{{N}_{h}} \end{array}} \right) $, and $\mathcal{V }= \left( {\scriptsize \begin{array}{@{}c@{}} \displaystyle ({\gamma }_{h}+{\mu }_{h}){I}_{h}\\ \displaystyle ({\gamma }_{v}+{\mu }_{v}){I}_{v} \end{array}} \right) $. Hence, $\mathbf{F}= \left( {\scriptsize \begin{array}{@{}cc@{}} \displaystyle 0&\displaystyle {\beta }_{vh}\\ \displaystyle {\beta }_{hv}\cdot \frac{{N}_{v}}{{N}_{h}} &\displaystyle 0 \end{array}} \right) $, and $\mathbf{V }= \left( {\scriptsize \begin{array}{@{}cc@{}} \displaystyle {\gamma }_{h}+{\mu }_{h}&\displaystyle 0\\ \displaystyle 0&\displaystyle {\gamma }_{v}+{\mu }_{v} \end{array}} \right) $. The next generation matrix, **G**, is given as follows.

\begin{eqnarray*}\mathbf{G}=\mathbf{F}{\mathbf{V }}^{-1}= \left( \begin{array}{@{}cc@{}} \displaystyle 0&\displaystyle \frac{{\beta }_{vh}}{{\gamma }_{v}+{\mu }_{v}} \\ \displaystyle m\cdot \frac{{\beta }_{hv}}{{\gamma }_{h}+{\mu }_{h}} &\displaystyle 0 \end{array} \right) , \end{eqnarray*}

where the term *m* is the vector to host ratio, which is defined by $m= \frac{{N}_{v}}{{N}_{h}} $. By solving the dominant eigenvalue of **G** (Van den Driessche & Watmough, 2002; Diekmann, Heesterbeek & Roberts, 2009; Cushing & Diekmann, 2016; Cushing & Diekmann, 2016), we derive the ${\mathcal{R}}_{0}$ of the model (2) in Eq. (3).

(3) \begin{eqnarray*}{\mathcal{R}}_{0}=\sqrt{ \left( \frac{m{\beta }_{hv}}{{\gamma }_{h}+{\mu }_{h}} \right) \cdot \left( \frac{{\beta }_{vh}}{{\gamma }_{v}+{\mu }_{v}} \right) }=\sqrt{m\cdot \frac{{\beta }_{hv}{\beta }_{vh}}{({\gamma }_{h}+{\mu }_{h})({\gamma }_{v}+{\mu }_{v})} }.\end{eqnarray*}

In Eq. (3), the first ratio under the square root, i.e., $ \frac{m{\beta }_{hv}}{{\gamma }_{h}+{\mu }_{h}} $, represents the number of vector infections caused by one infected host, and the second, i.e., $ \frac{{\beta }_{vh}}{{\gamma }_{v}+{\mu }_{v}} $, represents the number of host infections caused by one infected vector. The square root represents the geometric mean that takes the average number of secondary host (or vector) infections produced by a single infected host (or vector) (Van den Driessche, 2017).

### Effective reproduction number

During an epidemic, the susceptible individuals (*S*_*h*_ or *S*_*v*_) are gradually consumed, become infected and finally removed from the disease transmission cycle. The effective reproduction number, ${\mathcal{R}}_{\text{eff}}$, is the expected number of secondary cases produced by one typical infection joining in a population during its infectious period (Van den Driessche & Watmough, 2002). The ${\mathcal{R}}_{\text{eff}}$ is time-varying, which is denoted by ${\mathcal{R}}_{\text{eff}}(t)$ and sometimes ${\mathcal{R}}_{t}$ for the discretized situation (Ali, Kadi & Ferguson, 2013; Fraser, 2007; Cori et al., 2013), and ${\mathcal{R}}_{\text{eff}}$ quantifies the instantaneous transmissibility of the disease. By applying the approach in ‘Basic Reproduction Number’, the next generation matrix, **G**, is given by

\begin{eqnarray*}\mathbf{G} & = & \mathbf{F}{\mathbf{V }}^{-1}= \left( \begin{array}{@{}cc@{}} \displaystyle 0&\displaystyle {\beta }_{vh} \frac{{S}_{h}}{{N}_{h}} \\ \displaystyle {\beta }_{hv}\cdot \frac{{S}_{v}}{{N}_{h}} &\displaystyle 0 \end{array} \right) \times \left( \begin{array}{@{}cc@{}} \displaystyle ({\gamma }_{h}+{\mu }_{h})^{-1}&\displaystyle 0\\ \displaystyle 0&\displaystyle ({\gamma }_{v}+{\mu }_{v})^{-1} \end{array} \right) & = & \left( \begin{array}{@{}cc@{}} \displaystyle 0&\displaystyle \frac{{S}_{h}}{{N}_{h}} \cdot \frac{{\beta }_{vh}}{{\gamma }_{v}+{\mu }_{v}} \\ \displaystyle m\cdot \frac{{S}_{v}}{{N}_{v}} \cdot \frac{{\beta }_{hv}}{{\gamma }_{h}+{\mu }_{h}} &\displaystyle 0 \end{array} \right) . \end{eqnarray*}

Then, we derive ${\mathcal{R}}_{\text{eff}}$ from **G**.

\begin{eqnarray*}{\mathcal{R}}_{\text{eff}}=\sqrt{m\cdot \frac{{\beta }_{hv}{\beta }_{vh}}{({\gamma }_{h}+{\mu }_{h})({\gamma }_{v}+{\mu }_{v})} \cdot \frac{{S}_{h}}{{N}_{h}} \cdot \frac{{S}_{v}}{{N}_{v}} }={\mathcal{R}}_{0}\sqrt{ \frac{{S}_{h}}{{N}_{h}} \cdot \frac{{S}_{v}}{{N}_{v}} }, \end{eqnarray*}

where ${\mathcal{R}}_{0}$ is given in Eq. (3). We further define the susceptibilities of hosts (${\mathcal{S}}_{\text{h}}$) and vectors (${\mathcal{S}}_{\text{v}}$) by

(4) \begin{eqnarray*}{\mathcal{S}}_{\text{h}}= \frac{{S}_{h}}{{N}_{h}} ,\text{and} {\mathcal{S}}_{\text{v}}= \frac{{S}_{v}}{{N}_{v}} .\end{eqnarray*}

In the epidemic model (2), we have *S*_*h*_, *I*_*h*_, *R*_*h*_ ⩽ *N*_*h*_, and *S*_*v*_, *I*_*v*_, *R*_*v*_ ⩽ *N*_*v*_. Therefore, $0&les; {\mathcal{S}}_{\text{h}},{\mathcal{S}}_{\text{v}}&les; 1$. Henceforth, the effective reproduction number is in Eq. (5).

(5) \begin{eqnarray*}{\mathcal{R}}_{\text{eff}}={\mathcal{R}}_{0}\sqrt{{\mathcal{S}}_{\text{h}}{\mathcal{S}}_{\text{v}}}.\end{eqnarray*}

Since $0&les; {\mathcal{S}}_{\text{h}},{\mathcal{S}}_{\text{v}}&les; 1$, we have ${\mathcal{R}}_{\text{eff}}&les; {\mathcal{R}}_{0}$. Furthermore, (i) for most of the vector-borne diseases, the vector’s lifespan is much shorter than host’s lifespan, e.g., mosquito’s lifespan is around 7 to 60 days and tick’s lifespan is around 3 months to 3 years, and (ii) the infected vectors do not recover. These two facts will lead to an outcome in model (2) that class *R*_*v*_ = 0 and class *I*_*v*_ is extremely small and almost zero. Therefore, for simplicity, we set ${\mathcal{S}}_{\text{v}}=100\text{%}$ for the remaining parts of this study.

Importantly, the relationship between ${\mathcal{R}}_{0}$ and ${\mathcal{R}}_{\text{eff}}$ in Eq. (5) holds whenever the transmission path of the vector-borne pathogens is from a host to a host via a vector.

### An example of the yellow fever epidemic in Luanda 2015–2016

**Figure 2:**
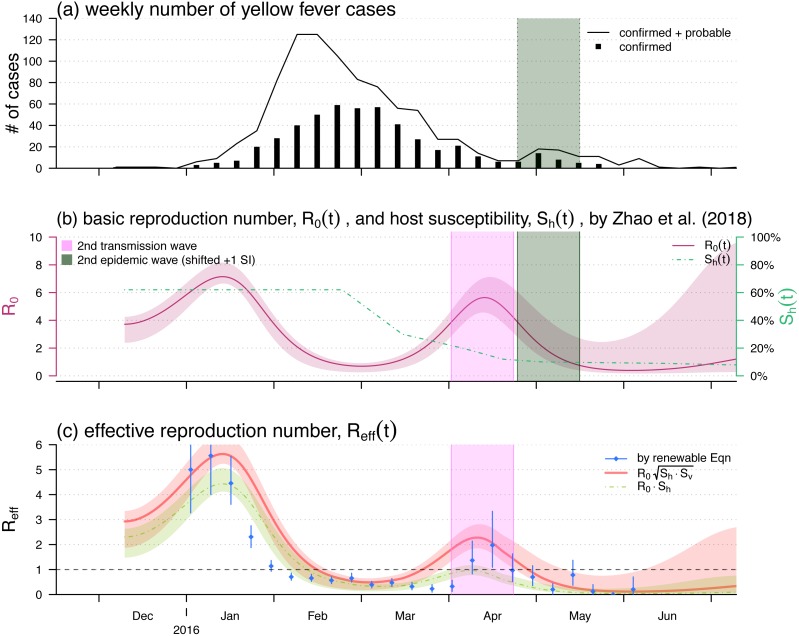
The yellow fever (YF) epidemic and reproduction number estimation in Luanda, Angola from 2015 to 2016. (A) The weekly number of YF cases time series, the second (minor) epidemic wave is shaded in grey. Panel (B) The reproduced time-varying basic reproduction number (purple curve), ${\mathcal{R}}_{0}(t)$, by using the reconstruction framework in Zhao et al. (2018b), and the time-varying human hosts’ susceptibility (green dashed line), ${\mathcal{S}}_{\text{h}}(t)$. The second transmission wave is highlighted in light purple by shifting one YF’s serial interval (SI), averagely 23 days Wu et al. (2016). The shedding area represent the 95% confidence intervals (CI). Panel (C) shows the effective reproduction numbers calculated (or estimated) by three different approaches, i.e., the $\mathcal{R}(t)$ by renewable equation (blue dots and bars) in Eq. (7), the product of ${\mathcal{R}}_{0}\sqrt{{\mathcal{S}}_{\text{h}}{\mathcal{S}}_{\text{v}}}$ (red bold curve) in Eq. (5), and the product of ${\mathcal{R}}_{0}{\mathcal{S}}_{\text{h}}$ (green dashed curve) in Eq. (1). The bars and the shedding areas represent the 95% CIs.

To illustrate the relationship between ${\mathcal{R}}_{0}$ and ${\mathcal{R}}_{\text{eff}}$ in Eq. (5), we compared different calculations or estimations of the effective reproduction number. We adopted the recent yellow fever (YF) outbreak in Luanda, Angola from 2015 to 2016 as a case study (Zhao et al., 2018b). The YF cases time series in Luanda and the local vaccination coverage were obtained from the situation reports released by the African Health Observatory (AHO) (WHO, 2017). Similar to the WHO (WHO, 2017) and previous literature (Kraemer et al., 2017; Zhao et al., 2018b), both probable and confirmed cases were grouped together, and were considered as the “YF cases” for further analyses (Fig. 2A).

We intend to demonstrate that the relationship between ${\mathcal{R}}_{0}$ and ${\mathcal{R}}_{\text{eff}}$ in Eq. (5) is more proper for pinpoint the epidemic control threshold of the vector-borne diseases than that in Eq. (1). To do so, we treat the instantaneous reproduction number, ${\mathcal{R}}_{t}$, calculated by the serial interval (SI) approach as the true effective reproduction number. The calculation of ${\mathcal{R}}_{t}$ is introduced in detail in ‘Instantaneously Reproduction Number Estimation by Renewable Equation’. After we find ${\mathcal{R}}_{t}$ series, we compare the calculations of the ${\mathcal{R}}_{\text{eff}}$s by using Eq. (1) or Eq. (5) to the ${\mathcal{R}}_{\text{eff}}$. The focus of this part is to compare the two relationships between ${\mathcal{R}}_{0}$ and ${\mathcal{R}}_{\text{eff}}$ described in Eqs. (1) and (5), and to identify which one is more proper for pinpoint the epidemic control threshold of the vector-borne diseases.

Note that although the formulation of ${\mathcal{R}}_{0}$ in model (2) is different from that in the YF epidemic model in Zhao et al. (2018b), the relationship between ${\mathcal{R}}_{0}$ and ${\mathcal{R}}_{\text{eff}}$ explored in Eq. (5) holds regardless of the complicity of the epidemic model.

The reproduction number, ${\mathcal{R}}_{0}(t)$ and ${\mathcal{R}}_{\text{eff}}(t)$, reconstruction approach proposed in Zhao et al. (2018b) is concisely introduced in ‘Reconstruction of the Reproduction Numbers from Compartmental Model’. The instantaneous (effective) reproduction number, $\mathcal{R}(t)$ or ${\mathcal{R}}_{t}$ estimation is described in details in ‘Instantaneously Reproduction Number Estimation by Renewable Equation’. We compared the two forms of effective reproduction number, ${\mathcal{R}}_{\text{eff}}(t)$, as in Eqs. (1) and (5) with the estimation by using the renewable equation in Eq. (7). To summary, Table 2 listed the relevant notations in this section as well as the remains of this study.

**Table 2:** Summary table of the reproduction numbers’ and susceptibilities’ notations. All of the variables listed here are unit-free.

Notation	Interpretation	Formulation	Remark
${\mathcal{R}}_{0}(t)$	Basic reproduction number	Eq. (3)	Reconstructed, ‘Reconstruction of the Reproduction Numbers from Compartmental Model’
${\mathcal{S}}_{\text{h}}(t)$	Susceptibility of hosts	Eq. (4)	Approximated, ‘Reconstruction of the Reproduction Numbers from Compartmental Model’
${\mathcal{S}}_{\text{v}}$	Susceptibility of vectors	Eq. (4)	Fixed to be 100%
${\mathcal{R}}_{\text{eff}}(t)$	Effective reproduction number	Eq. (1) or Eq. (5)	Calculated, ‘Reconstruction of the Reproduction Numbers from Compartmental Model’
$\mathcal{R}(t)$ or ${\mathcal{R}}_{t}$	Instantaneous (effective) reproduction number	Eq. (7)	Estimated, ‘Instantaneously Reproduction Number Estimation by Renewable Equation’

#### Reconstruction of the reproduction numbers from compartmental model

We reproduce the time-varying basic reproduction number, ${\mathcal{R}}_{0}(t)$, by using the compartmental model and reconstruction approach proposed in Zhao et al. (2018b). Here, we introduce the major reconstruction framework concisely, detailed procedures can be found in Zhao et al. (2018b). The same framework was also implemented to study other infectious diseases (He, Ionides & King, 2009; Ionides, Bretó & King, 2006; Shaman & Karspeck, 2012; Earn et al., 2012; Gao et al., 2016).

The reconstructed ${\mathcal{R}}_{0}(t)$ is in the form of an exponential cubic spline function varying over the YF epidemic period, i.e., from December 2015 to June 2016. The shape of the cubic spline function are controlled by the number of nodes and value of each node. We set the nodes are evenly distributed over the YF epidemic period. The ${\mathcal{R}}_{0}(t)$ cubic spline function is estimated based on the maximal likelihood framework. We treat the compartmental model simulated number of cases time series as the theoretical case numbers, denoted by *Z*_*i*_ for the *i*th week in the epidemic period. Note that the *Z*_*i*_s are from the underlying time-dependent version of model (2). By contrast, the number of observed YF cases time series, denoted by *C*_*i*_ for the *i*th week, are regarded as random samples from a negative binomial (NB) process determined by the theoretical case numbers. We assume the observation noise follows an over-dispersed Poisson distribution (Bretó et al., 2009), and in particular, the *C*_*i*_s follow a Poisson process determined by *Z*_*i*_s. Furthermore, the rate of the Poisson process is considered to be a Gamma random variable, and thus, this leads to a NB process (Lin et al., 2018). The probability framework is described in Eq. (6).

(6) \begin{eqnarray*}{C}_{i}\sim \text{NB} \left( \text{size}= \frac{1}{\tau } ,\text{probability}= \frac{1}{1+\tau {Z}_{i}} \right) , \text{with mean}={Z}_{i} \mathrm{{\XMLAMP}} \text{variance}={Z}_{i}(1+\tau {Z}_{i}),\end{eqnarray*}

where the term *τ* is an over-dispersion parameter of the NB process that needs to be estimated. Thus, the overall log-likelihood value can be calculated by summing up to all log-probabilities of all *i*s during the entire YF epidemic period. Therefore, the reconstructed ${\mathcal{R}}_{0}(t)$ can be estimated by finding the number of nodes and values of nodes (of the cubic spline function) with the “best fitting performance”. We evaluate the fitting performance of the reconstructed ${\mathcal{R}}_{0}(t)$ by measuring the trade-off between the goodness-of-fit (in term of the log-likelihood) and the complexity of the model structure (in term of the number of parameters to be estimated). As in Zhao et al. (2018b), the Bayesian information criterion (BIC) is employed to evaluate the fitting performance. By using the likelihood profile approach (He, Ionides & King, 2009; He et al., 2017; Zhao et al., 2018a; Barndorff-Nielsen & Cox, 1994; Ionides, Bretó & King, 2006), in which the profile of maximum log likelihood was calculated as a function of the model parameter, we estimated the 95% confidence interval (CI) of the reconstructed ${\mathcal{R}}_{0}(t)$. The simulation outcomes can be found in Fig. 3 of Zhao et al. (2018b).

By using the local YF vaccination coverage, the data were publicly available via the African Health Observatory (WHO, 2017) as well as adopted in Zhao et al. (2018b), we approximated the time-varying population susceptibility, ${\mathcal{S}}_{\text{h}}(t)$, as shown in Fig. 2B. The approximation is difference between the pre-existed population susceptibility, calculated by 1 minus the pre-existed YF-protection rate, and the time-varying local vaccination coverage.

Given ${\mathcal{R}}_{0}(t)$ and ${\mathcal{S}}_{\text{h}}(t)$, we calculated two different forms of the time-varying effective reproduction number by using the Eqs. (1) and (5) with ${\mathcal{S}}_{\text{v}}=1$ fixed.

#### Instantaneously reproduction number estimation by renewable equation

The transmissibility of YF can be quantified by calculating the instantaneous (effective) reproduction number, $\mathcal{R}(t)$ and ${\mathcal{R}}_{t}$ for discrete scenarios, defined as the expected number of secondary cases generated by a single infectious individual during the infectious periods at time *t*. We estimated the $\mathcal{R}(t)$ from the YF cases time series by using the serial interval (SI) approach proposed by Wallinga & Teunis (2004). The SI, in the epidemiology of infectious diseases, is the period of time between successive cases in a chain of transmission (Porta, 2014; Fine, 2003). If we know the distribution of the inter-arrival time of patients arrived at a clinic, we may simulate the sequence of patients arrivals. Similarly, if we know the distribution of SI, we may simulate the sequence of infections, adding that one primary infection could lead to a number of, which is determined by the reproduction number, secondary infections. Reversely, if we know the distribution of SI and the cases time series, we can reconstruct the reproductive number backwardly. This SI approach was extended by Forsberg White & Pagano (2008), Katriel et al. (2011), Ali, Kadi & Ferguson (2013), Fraser (2007), Cori et al. (2013) and Wallinga & Lipsitch (2006), and also implemented to study several vector-borne diseases (Ferguson et al., 2016; Zhao et al., 2019a; Wu et al., 2016; Zhao et al., 2019b). Hence, the time-varying $\mathcal{R}(t)$ is estimated from the renewal equation in Eq. (7).

(7) \begin{eqnarray*}\mathcal{R}(t)= \frac{x(t)}{\int \nolimits \nolimits _{0}^{\infty }w(k)x(t-k)\,\text{d}k} ,\end{eqnarray*}

where *x*(*t*) is the YF incidence rate at time *t*. The convolution term $\int \nolimits _{0}^{\infty }w(k)x(t-k)\text{d}k$ is the measurement of the total infectiousness at time *t*. The term *w*(*k*) is the YF SI distribution that describes the distribution of the infectiousness during the period of infection. We adopted the same approach, similar methods were also implemented in Zhao et al. (2019c), Zhao et al. (2019d), Cowling et al. (2009) and Ferguson et al. (2016), to find SI, *w*(*k*), and epidemiology parameter setting as in Wu et al. (2016) as well as the references mentioned in it (Johansson et al., 2012; Johansson et al., 2010), and we had the numerical estimation of *w*(*k*) with the mean SI of 23 days.

We estimated the $\mathcal{R}(t)$ of YF between January and May of 2016. After this time period, weeks of zero confirmed case appeared. The 95% confidence intervals (CI) were estimated based on the Gamma priors of each $\mathcal{R}(t)$ (Ali, Kadi & Ferguson, 2013; Cori et al., 2013).

## Results

**Figure 3:**
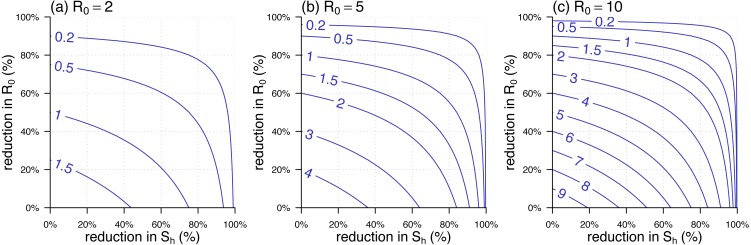
The contour plot of the effective reproduction number, ${\mathcal{R}}_{\text{eff}}$, against the percentage reductions in ${\mathcal{S}}_{\text{h}}$ and ${\mathcal{R}}_{0}$. (A –C) The scenarios of highest ${\mathcal{R}}_{0}=2$, ${\mathcal{R}}_{0}=5$, and ${\mathcal{R}}_{0}=10$, respectively. The labels on the blue curves show the values of ${\mathcal{R}}_{\text{eff}}$. The horizontal axis is the percentage reduction in ${\mathcal{S}}_{\text{h}}$. The vertical axis is the percentage reduction in ${\mathcal{R}}_{0}$ that is 1 minus the ratio of the reduced ${\mathcal{R}}_{0}$ over its original (or highest) value, i.e., the value in the panel label.

The analytic formula of ${\mathcal{R}}_{\text{eff}}$ was given in Eq. (5). Since in many situations, the ${\mathcal{R}}_{0}(t)$ is time-varying, we defined the percentage reduction in ${\mathcal{R}}_{0}$ as 1 minus the ratio of the reduced ${\mathcal{R}}_{0}$ over its original highest value, usually the basic reproduction number during the initial outbreak. Similarly, the percentage reduction in ${\mathcal{S}}_{\text{h}}$ can be defined as 1 minus the host susceptibility. We noted that both percentage reductions in ${\mathcal{S}}_{\text{h}}$ and ${\mathcal{R}}_{0}$ ranged from 0 to 1. The relationship of ${\mathcal{R}}_{\text{eff}}$ against the percentage reductions in ${\mathcal{S}}_{\text{h}}$ and ${\mathcal{R}}_{0}$ was shown in Fig. 3. Figs. 3(A)–3(C) were the scenarios of the highest ${\mathcal{R}}_{0}=2$, ${\mathcal{R}}_{0}=5$, and ${\mathcal{R}}_{0}=10$ respectively. The range of ${\mathcal{R}}_{0}$ from 2 to 10 covers most of the vector-borne diseases’ basic reproduction numbers during the initial outbreak.

We used the 2015–2016 YF outbreak in Luanda to demonstrate the effective reproduction number formula in Eq. (5). The YF epidemic was shown in Fig. 2A. Subsequent to the (first) major epidemic wave that peaked in the February of 2016, we observed a second minor wave that followed the major wave, which peaked in May. We showed the reconstructed ${\mathcal{R}}_{0}(t)$ and approximated ${\mathcal{S}}_{\text{h}}(t)$ reproduced from Zhao et al. (2018b) in Fig. 2B. The local human susceptibility, ${\mathcal{S}}_{\text{h}}(t)$, was decreasing by the end of February and ended up less than 7% due to the timely mass vaccination campaign (Shearer et al., 2017; Wu et al., 2016; Zhao et al., 2018b; Kraemer et al., 2017). We found two peaks in the reconstructed ${\mathcal{R}}_{0}(t)$, of which the highest value was found to be 7.1 during the first wave that peaked in January. The second peak of ${\mathcal{R}}_{0}(t)$ occurred in the April of 2016 with the local maximal value of 5.6. We matched the second peak in ${\mathcal{R}}_{0}(t)$, highlighted in purple, and the minor epidemic wave in the YF incidences, highlighted in grey, by one SI shift, i.e., 23 days averagely (Wu et al., 2016).

Figure 2C shows the effective reproduction number calculated or estimated by Eq. (1) or Eq. (5) or the renewable equation in Eq. (7). The (first) major transmission, i.e., ${\mathcal{R}}_{\text{eff}}(t)$, wave peaked in January of 2016 associated with the major epidemic wave that peaked in February (Fig. 2A). We found the ${\mathcal{R}}_{\text{eff}}(t)$ series based on ${\mathcal{R}}_{0}(t)$ and ${\mathcal{S}}_{\text{h}}(t)$ (in Fig. 2B) were (almost) synchronised, i.e., in-phase, with the estimated ${\mathcal{R}}_{\text{eff}}(t)$ (or $\mathcal{R}(t)$) series by the renewable equation. The highest ${\mathcal{R}}_{\text{eff}}(t)$ estimate by renewable equation was 5.5, and the same as the estimate by using Eq. (5) that was also 5.5, whereas the Eq. (1) version is 4.4. Similar to the trends of YF incidences and ${\mathcal{R}}_{0}(t)$, we also found a second minor wave in ${\mathcal{R}}_{\text{eff}}(t)$ around April, highlighted in purple. During this minor wave, the local maximal ${\mathcal{R}}_{\text{eff}}(t)$ estimate by renewable equation was 2.0 (95% CI [1.1–3.4]) that is larger than 1 significantly, the “ ${\mathcal{R}}_{0}\sqrt{{\mathcal{S}}_{\text{h}}{\mathcal{S}}_{\text{v}}}$” version was 2.3, and the “ ${\mathcal{R}}_{0}{\mathcal{S}}_{\text{h}}$” version is 0.9 ( <1).

**Figure 4:**
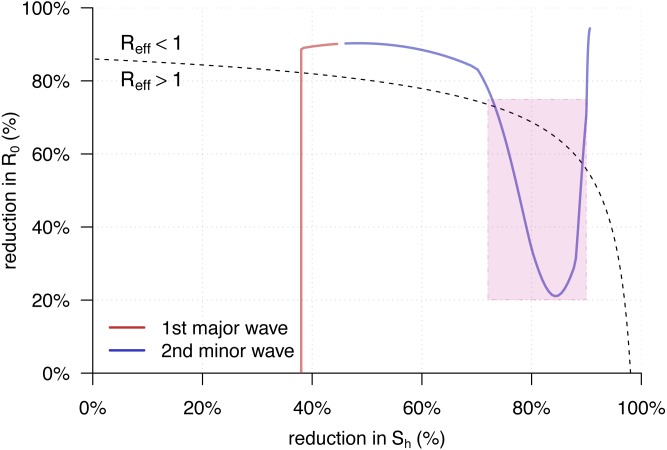
The trajectory of the ${\mathcal{R}}_{\text{eff}}(t)$ from Eq. (5) against the percentage reductions in ${\mathcal{S}}_{\text{h}}$ and ${\mathcal{R}}_{0}$ of the yellow fever (YF) epidemic in Luanda, Angola from 2015 to 2016. The horizontal and vertical axes have the same setting as in Fig. 3. Here, the original highest ${\mathcal{R}}_{0}=7.1$ as the same as in Fig. 2B. The black dashed curve represents the level of ${\mathcal{R}}_{\text{eff}}=1$. The area under the ${\mathcal{R}}_{\text{eff}}=1$ curve is for ${\mathcal{R}}_{\text{eff}}&gt; 1$, and those above is for ${\mathcal{R}}_{\text{eff}}&lt; 1$. The red trajectory represents the changing dynamics of ${\mathcal{S}}_{\text{h}}$ and ${\mathcal{R}}_{0}$ during the first major epidemic wave from December 2015 to February 2016. The purple trajectory represents the changing dynamics of ${\mathcal{S}}_{\text{h}}$ and ${\mathcal{R}}_{0}$ during the second minor epidemic wave from March to May 2016. The ${\mathcal{R}}_{\text{eff}}&gt; 1$ part during the second transmission wave, highlighted in purple in Fig. 2C, are marked in the purple rectangle.

Figure 4 showed the trajectory of the ${\mathcal{R}}_{\text{eff}}(t)$ from Eq. (5) against the percentage reductions in ${\mathcal{S}}_{\text{h}}$ and ${\mathcal{R}}_{0}$ of the YF epidemic in Luanda. Consistent with the observations in Fig. 2C, we found both of the two transmission waves of YF ${\mathcal{R}}_{\text{eff}}(t)$ moved across the disease control threshold, i.e., ${\mathcal{R}}_{\text{eff}}=1$. For the second transmission waves, marked in the purple rectangle, it “broke” the ${\mathcal{R}}_{\text{eff}}=1$ boundary and thus associated with the second (minor) YF epidemic wave in May of 2016, highlighted in grey in Fig. 2A.

## Discussion

In this work, a simple epidemic model (2) is developed to study the transmission dynamics of vector-borne diseases. We formulated the analytic form of the effective reproduction number, ${\mathcal{R}}_{\text{eff}}$, with respect to the basic reproduction number, ${\mathcal{R}}_{0}$, and the susceptibilities of the vector (${\mathcal{S}}_{\text{v}}$) and host (${\mathcal{S}}_{\text{h}}$) for vector-borne diseases in Eq. (5). The ${\mathcal{R}}_{\text{eff}}$ from Eq. (5) were compared with the ${\mathcal{R}}_{\text{eff}}$ of the classic airborne infectious disease in Eq. (1) as well as the estimation by the SI approach in Eq. (7). We re-visited the yellow fever (YF) outbreak in Luanda, and used this epidemic as an example to compare the ${\mathcal{R}}_{\text{eff}}$ calculation and estimation. Although there existed differences in the three ${\mathcal{R}}_{\text{eff}}$ series during the first transmission wave around January 2016 in Fig. 2, the ${\mathcal{R}}_{\text{eff}}$ values were roughly synchronised. However, for the second (minor) transmission wave around April 2016, the ${\mathcal{R}}_{\text{eff}}$s from Eq. (5) were consistent with the estimates from the renewable equation that were significantly larger than 1, the “ ${\mathcal{R}}_{0}{\mathcal{S}}_{\text{h}}$” appeared inconsistent with the formers and lower than 1. According to theoretical epidemiology (Earn et al., 2008; Brauer & Castillo-Chavez, 2001; Keeling & Rohani, 2011; Grenfell, Dobson & Moffatt, 1995), the condition that ${\mathcal{R}}_{\text{eff}}&lt; 1$ guarantees the disease under control. The ${\mathcal{R}}_{0}{\mathcal{S}}_{\text{h}}$ was calculated lower than 1 since the mid-February 2016, and this contradicted with the occurrence of the second YF epidemic wave in May. Therefore, the “ ${\mathcal{R}}_{0}{\mathcal{S}}_{\text{h}}$” form of effective reproduction number was demonstrated unqualified for measuring the transmissibility of a vector-borne disease. On the other hand, our derived ${\mathcal{R}}_{\text{eff}}={\mathcal{R}}_{0}\sqrt{{\mathcal{S}}_{\text{h}}{\mathcal{S}}_{\text{v}}}$, Eq. (5), matched the two waves of both YF incidences time series and the $\mathcal{R}(t)$ estimates by renewable equation well.

Different from the vector-free context, the ${\mathcal{R}}_{\text{eff}}={\mathcal{R}}_{0}\sqrt{{\mathcal{S}}_{\text{h}}{\mathcal{S}}_{\text{v}}}$ for the vector-borne diseases indicates that the disease control effectiveness, i.e., ${\mathcal{R}}_{\text{eff}}$, non-linearly depends on the control of the host’s susceptibility, ${\mathcal{S}}_{\text{h}}$. Figure 3 shows that the reduction in ${\mathcal{S}}_{\text{h}}$ is (relatively) less effective in reducing ${\mathcal{R}}_{\text{eff}}$ during the initial stage, i.e., from 0% onwards, and becomes more effective when the cumulative reduction of ${\mathcal{S}}_{\text{h}}$ grows. This finding suggests that directly reducing ${\mathcal{R}}_{0}$, via, e.g., vector elimination, avoiding exposure to vectors, improving treatment, etc., could be a more efficient option to control the vector-borne diseases, especially when the herd protection in the host population is difficult to build up.

Although the next generation matrix method in ‘Basic Reproduction Number’ is valid around a disease-free equilibrium (DFE) of model (2) (Van den Driessche & Watmough, 2002; Van den Driessche, 2017), the (asymptotic) stability of the endemic equilibrium (EE) will allow that the product of the ${\mathcal{R}}_{0}$ multiplying the susceptibility can be interpreted as ${\mathcal{R}}_{\text{eff}}$. The *B*_*v*_(*t*) can be treated as a constant, 〈*B*_*v*_〉, when we consider a sufficiently short period of time. Hence, during this short period of time, the EE of model (2) is asymptotically stable, and the Eq. (5) holds. More precisely, Eq. (5) follows a more general version as follows,

\begin{eqnarray*}{\mathcal{R}}_{\text{eff}}(t)={\mathcal{R}}_{0}(t)\sqrt{{\mathcal{S}}_{\text{h}}(t){\mathcal{S}}_{\text{v}}(t)}. \end{eqnarray*}

The global asymptotic stability (GAS) of EE can be further guaranteed as ${N}_{v}^{{^{\prime}}}={B}_{v}(t)-{\mu }_{v}{N}_{v}=0$ in model (2), and this leads to the condition that *B*_*v*_(*t*) = 〈*B*_*v*_〉 = *μ*_*v*_*N*_*v*_.

This work used the serial interval (SI) approach, i.e., the renewable equation, to estimate the instantaneous effective reproduction number, $\mathcal{R}(t)$, for further comparison. The estimates of $\mathcal{R}(t)$ depended on the choice of the distribution of SI, i.e., the *w*(*k*) in Eq. (7). Accounting for the initial susceptibility of 63% of the YF epidemic (WHO, 2017), Wu et al. (2016) estimated that the YF basic reproduction number of ${\mathcal{R}}_{0}=8.3$ (95% CI [6.8–9.7]) with mean SI of 23 days, and ${\mathcal{R}}_{0}=11.3$ (95% CI [8.7–13.8]) with mean SI of 32 days. Kraemer et al. (2017) estimated that ${\mathcal{R}}_{0}=7.6$ (95% CI [6.3–8.9]) with mean SI 15 days. We adopted the mean SI of 23 days as in Wu et al. (2016) in this work to estimate the $\mathcal{R}(t)$ series. Different (but reasonable) settings on SI will distinguish the second transmission wave (as highlighted in purple in Figs. 2C and 4) with ${\mathcal{R}}_{\text{eff}}&gt; 1$ indifferently. In addition, we note that slight changes in the choice of YF SI will not affect our main results.

This work intends to estimate and compare different forms of the time-varying effective reproduction number. We have adopted two different but widely used approaches, i.e., the maximal likelihood-based reconstruction and the SI, i.e., by the renewable equation, based estimation, which includes three different formations in Eqs. (1), (5) and (7). The maximum likelihood-based reconstruction method and the SI based estimation method are different in the calculation procedures and theoretical features. The maximum likelihood-based reconstruction relies on the mechanistic disease transmission model, e.g., model (2), and thus, it follows a biologically reasonable model structure. It is able to disentangle the changing dynamics of susceptibility, ${\mathcal{S}}_{\text{h}}(t)$, and the basic reproduction number, ${\mathcal{R}}_{0}(t)$, based the number of cases time series and other reasonable epidemiological settings. Hence, we adopted this approach to find both ${\mathcal{S}}_{\text{h}}(t)$ and ${\mathcal{R}}_{0}(t)$, and further calculate the ${\mathcal{R}}_{\text{eff}}(t)$ in two difference forms. The SI based (renewable equation based) estimation method is to calculate descriptive statistics by nature. By directly using the number of disease cases time series and the knowledge (distribution) of SI, the $\mathcal{R}(t)$ can be estimated straightforwardly.

To derive the ${\mathcal{R}}_{\text{eff}}$ in Eq. (5), we used the next generation matrix approach in ‘Effective Reproduction Number’ and considered the transition “from host to vector to host” as two generations, which is consistent with (Gao et al., 2016; Tang et al., 2016; Zhao et al., 2018b; Champagne et al., 2016; d Pinho et al., 2010; Musa et al., 2019; Wang et al., 2012). As also remarked in Brauer et al. (2016) and Van den Driessche (2017), some other studies treated the same transition “from host to vector to host” as a single combined generation (Tennant & Recker, 2018; Chowell et al., 2007; Kucharski et al., 2016; Towers et al., 2016; Mideo & Day, 2008). Although the two choices have the same threshold value and follow the same mathematical criteria to judge the stability of compartmental models, to be aware of their difference is crucial. We remark that if considering the aforementioned transition as a single generation, the basic reproduction number would be the square of the ${\mathcal{R}}_{0}$ in Eq. (3). In this case, the effective reproduction number is ${\mathcal{R}}_{\text{eff}}={\mathcal{R}}_{0}{\mathcal{S}}_{\text{h}}{\mathcal{S}}_{\text{v}}$. In our YF epidemic example, we demonstrated that the misuse of the Eq. (1) is likely to cause misleading or contradictory outcomes in studying the vector-borne diseases outbreak. The two forms of the reproduction numbers have different biological interpretations due to the different definitions of generations, nevertheless one can be transformed to the other.

Our modelling study, specially the derived “ ${\mathcal{R}}_{\text{eff}}={\mathcal{R}}_{0}\sqrt{{\mathcal{S}}_{\text{h}}{\mathcal{S}}_{\text{v}}}$” relationship, has limitations mainly due to the model settings and structures. As stated in the analysis parts, the relationship holds on the condition that the transmission paths remain from-host-to-vector and from-vector-to-host. Hence, when direct transmission occurs, e.g., sexual transmission between hosts in Zika virus (Gao et al., 2016), the ${\mathcal{R}}_{\text{eff}}\not = {\mathcal{R}}_{0}\sqrt{{\mathcal{S}}_{\text{h}}{\mathcal{S}}_{\text{v}}}$. However, since the sexual transmission merely contributes very minor infections, it can be ignored in scale, and thus ${\mathcal{R}}_{\text{eff}}\approx {\mathcal{R}}_{0}\sqrt{{\mathcal{S}}_{\text{h}}{\mathcal{S}}_{\text{v}}}$. The numerical results and estimates in this work are calculated with ${\mathcal{S}}_{\text{v}}=1$ fixed. This ${\mathcal{S}}_{\text{v}}=1$ is based on two facts that the vector’s lifespan is much shorter than host’s lifespan; and the infected vectors do not recover (for most of the vector-borne diseases). These two facts will lead to an outcome in model (2) that class *R*_*v*_ = 0 and class *I*_*v*_ is remarkably small and almost zero. As the matter of fact, *I*_*v*_, i.e., the prevalence of disease in vectors, is likely to increase when the pathogen is extremely infectious and the vertical transmission accounts. Therefore, the surveillance on the disease prevalence in vectors would be helpful for calculating the ${\mathcal{R}}_{\text{eff}}$.

## Conclusions

We formulate the analytic form of the ${\mathcal{R}}_{\text{eff}}={\mathcal{R}}_{0}\sqrt{{\mathcal{S}}_{\text{h}}{\mathcal{S}}_{\text{v}}}$ for the vector-borne diseases. We demonstrate the ${\mathcal{R}}_{\text{eff}}$ formulation is consistent with the estimates of the 2015–2016 yellow fever outbreak in Luanda, and distinguishes the second minor epidemic wave significantly. We remark that it is important to be aware of whether one or two generations is considered for the transition “from host to vector to host” in the infectious diseases modelling studies.

##  Supplemental Information

10.7717/peerj.8601/supp-1 Supplemental Information 1 R code

10.7717/peerj.8601/supp-2 Supplemental Information 2 Raw data

## Additional Information and Declarations

### Data Availability

The following information was supplied regarding data availability: This is a methodology paper, and the method with examples is explained in the text. Data and code are available in the Supplemental Files.
